# Perceived exertion, postural control, and muscle recruitment in three different quadruped exercises performed by healthy women

**DOI:** 10.3389/fphys.2022.948469

**Published:** 2022-08-19

**Authors:** Patrícia Cardoso Clemente, Luane Landim de Almeida, Eduardo José Danza Vicente, Diogo Simões Fonseca, Victor Hugo Souza, Diogo Carvalho Felício, Marco Antonio Cavalcanti Garcia

**Affiliations:** ^1^ Programa de Pós-Graduação em Ciências da Reabilitação e Desempenho Físico Funcional, Faculdade de Fisioterapia, Universidade Federal de Juiz de Fora, Juiz de Fora, Minas Gerais, Brazil; ^2^ Faculdade de Ciências Médicas e da Saúde de Juiz de Fora (SUPREMA), Juiz de Fora, Minas Gerais, Brazil; ^3^ Departamento de Fisioterapia Cardiorrespiratória e Musculoesquelética, Faculdade de Fisioterapia, Universidade Federal de Juiz de Fora, Juiz de Fora, Minas Gerais, Brazil; ^4^ Department of Neuroscience and Biomedical Engineering, Aalto University, School of Science, Espoo, Finland; ^5^ Grupo de Neuro Biomecânica, Faculdade de Fisioterapia, Universidade Federal de Juiz de Fora, Juiz de Fora, Minas Gerais, Brazil; ^6^ Departamento de Fisiologia, Instituto de Ciências Biológicas, Universidade Federal de Juiz de Fora, Juiz de Fora, Minas Gerais, Brazil

**Keywords:** low back pain, postural balance, physical fitness, exercise movement techniques, abdominal muscles

## Abstract

Although quadruped exercises (QE) have been a part of rehabilitation and sports programs, there is no clarity on how these exercises challenge the musculoskeletal system. Therefore, this cross-sectional study investigated the perceived exertion, postural demands, and muscle recruitment profiles imposed by three QE postures. Surface electromyographic (sEMG) signals were recorded from *transverse abdominis*, *longissimus dorsi*, *multifidus*, and *iliocostalis lumborum* from 30 sedentary healthy women, bilaterally. They performed the classic quadruped exercise (CQ), a variation with shoulder flexion (FQ), and the homolateral quadruped (HQ). Borg scores (BS) and the center of pressure (CoP) from the palmar statokinesiogram were also recorded. Surface EMG signals were normalized using the myoelectric activity recorded from two other postures while performing isometric voluntary contractions (IVC). Results were analyzed using one- (CoP) and three-way (sEMG data) ANOVA with Bonferroni post hoc tests (*α* = 0.05). The Borg scale was analyzed using the Friedman test. The CQ provided lower BS and CoP than HQ (*p* < 0.05), followed by a higher sEMG activity (∼51% of IVC) than FQ (∼47% of IVC; *p* = 0.53) and HQ (∼44% of IVC; *p* = 0.01). In turn, HQ provided greater BS (*p* > 0.05) than CQ and FQ. The results suggested that the HQ was the most challenging exercise regarding CoP and BS, although CQ presented a higher symmetrical sEMG activity. Since QE are often prescribed in exercise programs, specific knowledge of the characteristics of each QE makes prescribing safer and more efficient.

## Introduction

Spinal stabilization exercises have been usually adopted to treat and prevent low back pain and promote the physical performance of athletes and non-athletes (Graham, 2009; Manchikanti et al., 2014; Knox et al., 2017). Quadruped exercises (QE) are a sort of these activities, which are essentially featured by taking a four-support posture with upper and lower limbs held entirely or partially on the ground (Graham, 2009; Kelly et al., 2016). QE are clearly understood as leading to the dynamic stabilization of the pelvic and scapular girdles by favouring the axial stretching of the vertebral column and promoting the strengthening of the abdominal and paravertebral muscles (Ekstrom et al., 2007). Therefore, QE have been widely discussed in the literature due to their relevance in rehabilitation, sports, and health exercises (Ekstrom et al., 2007; Kelly et al., 2016; Shah et al., 2020).

We can observe several variations in the QE execution. QE are also commonly named as *leg and arm pull front*, *bird dog*, and *hip and shoulder extension in a four-position stance* due to the many possibilities in the positions adopted (Chou et al., 2007; Lunes et al., 2010; Kelly et al., 2016). Consequently, different body postures, with or without external support, and various forms of performance (symmetrical or asymmetrical, bilateral or homolateral movements) comprise some QE properties. For instance, the functional quadruped (FQ) exercise, which requires a maximal extension of the upper limb and contralateral lower limb extension to 0° and maximum plantar flexion, is part of the Klein-Vogelbach (1990) functional kinetic method. Therefore, the movement proposed in this posture is suggested to be a functional movement linked to gait, where the upper limb swings in phase with the contralateral lower one. Even so, although previous studies have provided some insights into the electromyographic pattern of various trunk muscles during the execution of different QE postures (Calatayud et al., 2017), others have failed to clarify their suitability for preventing sports injuries (Blasimann et al., 2018) and treating low back pain (Gupta and Alok, 2020).

Therefore, although we conjecture that the diversity of QE postures (Youdas et al., 2014; Kelly et al., 2016) may offer trainers and physical therapists the opportunity to adapt them to different groups, there seems to be a lack of understanding of how they can challenge their practitioners. Hence, it sounds imperative not only to characterize QE from their muscle recruitment pattern but also from other parameters which can subsidize therapists and trainers in prescribing these exercises. Thus, additionally to the perceived effort and muscle recruitment, which can be determined by using the Borg scale and the myoelectric activity, respectively, but also to comprehend how challenging each posture can be, can help clarify the appropriateness of these exercises. As for the challenges imposed by QE in postural control, it is our understanding that the exploration using the center of pressure excursion area from the base of support may be a suited approach for this purpose. However, to the best of our knowledge, no previous research was able further to characterize QE postures in the light of those parameters. Therefore, the present study aimed to investigate the perceived exertion, postural demands, and muscle recruitment profiles imposed by three traditional QE postures in healthy women.

## Material and methods

### Participants

The sample size was estimated using the software GPower (version 3.1.9 Düsseldorf, Germany) (Faul et al., 2007). The estimation parameters for a F test family were: Effect size f of 0.25, power = 0.80 and 5% (*α* = 0.05) of significance level, which allowed us to set a minimum sample size of 29 volunteers. All participants were right-handed, according to Oldfield’s inventory (Oldfield, 1971), free of neurological and motor disorders, and classified as sedentary or insufficiently active according to the International Physical Activity Questionnaire (IPAQ) in its short version (Matsudo et al., 2001). The exclusion criteria were as follows: disabling low back pain in the last 12 months, a herniated disc, scoliosis, neurological or infectious diseases, lower limb dysmetria (lower limbs) and/or upper limbs (verified by the physical examination performed by the researchers), back pain during the day of data recording, cancer, pregnancy, surgical interventions in the spine, skin lesions at the electrode fixation sites, and failure to perform the three different postures studied before data recording. The local ethics committee approved the study (Universidade Federal de Juiz de Fora, Minas Gerais, Brazil; n. 2.634.323) in conformity with the Declaration of Helsinki and conducted during the year 2019. All participants were informed about the characteristics of the study and signed the informed consent form before participation in the experimental protocol.

### Instrumentation and procedures

The QE postures were investigated only from the maintenance of the right (dominant) hand on the ground and under isometric contraction conditions, and all the data were recorded in a single acquisition session. The modified Borg scale (levels 1–10) was used at the end of the three repetitions of each QE posture to obtain the perceived exertion index from each participant (Borg, 1998).

A pressure plate (FootWork, France; A/D conversor: 16-bit; sampling frequency: 150 Hz; 400 mm × 400 mm) was used to record the time series of the center of pressure (CoP) from the dominant hand on the ground support to evaluate how challenging each of the QE adopted was to maintaining the postural stability. The elliptical CoP area from the statokinesiogram was used to characterize QE and to measure how much the quadrupedal postural stability control challenged the palmar support base. The pressure platform records the anterior-posterior and middle-lateral displacements, thus inferring the stability level of the adopted posture.

We recorded the surface myoelectric activity (sEMG) of four different trunk muscles (*transverse abdominis* [TA]; *iliocostalis lumborum* [IC]; *longissimus dorsi* [LD]; and *multifidus* [MD], bilaterally). The sEMG surface electrodes (*Solidor®*, Medico Electrodes International Ltd., Uttar Pradesh, India; Ag-AgCl; 1 cm diameter) were placed on the corresponding muscle belly in a bipolar configuration with an interelectrode distance of 20 mm, in agreement with SENIAM recommendations (Hermens et al., 2000) and Knox et al. (2017). The reference electrode was placed over the cervical prominence C7. The skin was shaved and cleaned with alcohol and neutral soap before placing the electrodes. The sEMG signals were digitized (EMG System do Brasil Ltda, São José dos Campos, Brazil; model: 810C; gain: 2000, sampling frequency: 2.0 kHz per channel; filter: band-pass fourth order Butterworth: 20–500 Hz; A/D conversor: 16 Bits) and recorded using the software EMGLab V1.1 (version 2012; Lynx Tecnologia Eletrônica Ltda, São Paulo, SP, Brazil). Besides adopting the SENIAM recommendations to reduce the risks of *bias* of the sEMG signal, the recording system was powered by batteries without any other connection to the electrical supply.

**FIGURE 1:**
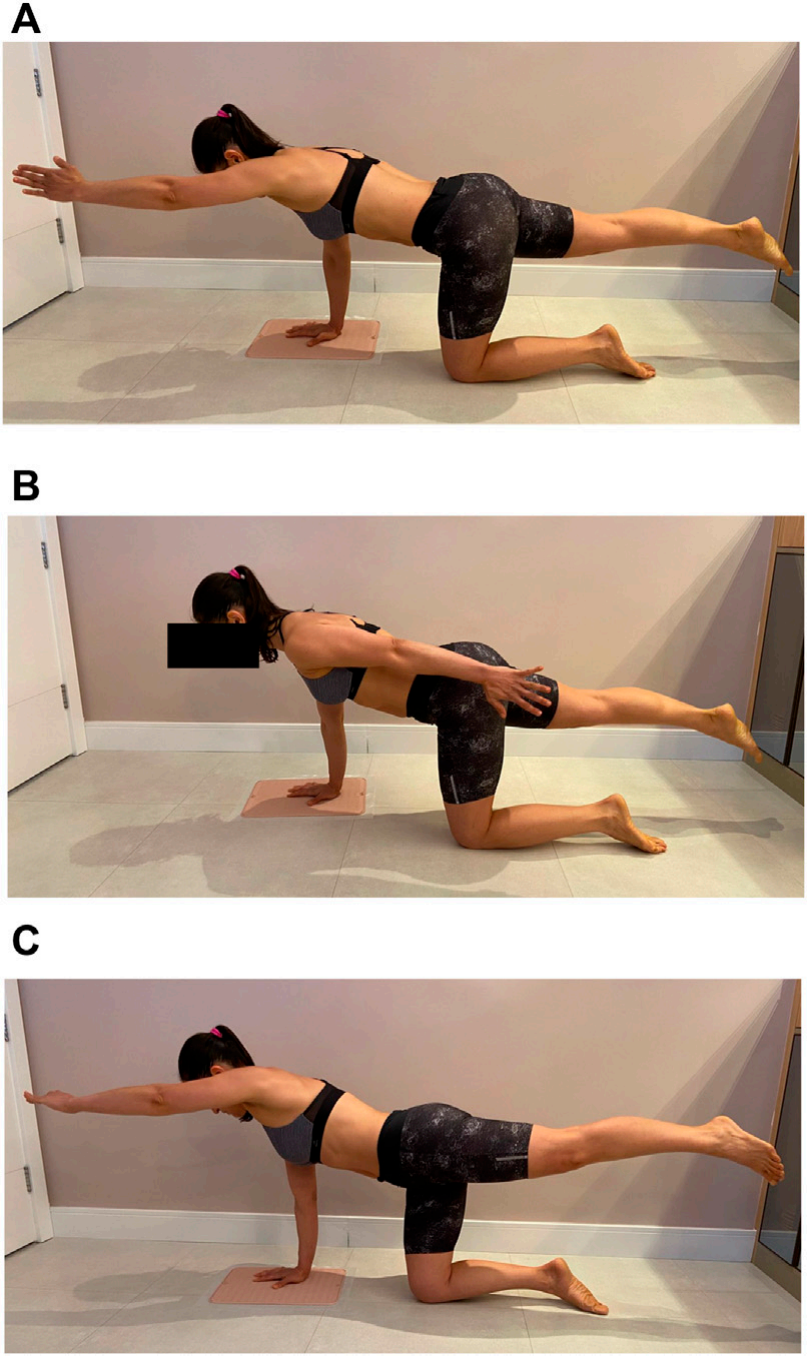
Final positions of the analyzed exercises. **(A)** Classic Quadruped **(B)** Functional Quadruped, and **(C)** Homolateral Quadruped.

**FIGURE 2:**
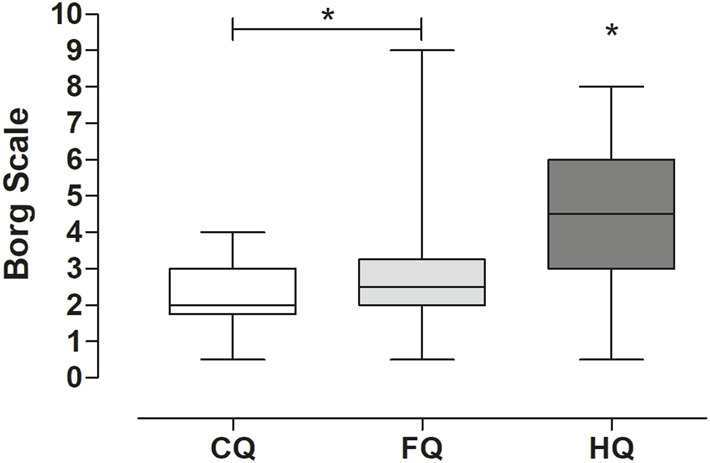
Results (medians and quartiles) from Borg scale for the three QE. The homolateral exercise (HQ) resulted in significant (*p < 0.05) higher levels of perceived effort in contrast to the other two (CQ and FQ).

Participants performed three different QE from a starting position (in a four-stance position, knees aligned at the width of the hip joint, upper limbs at 90° shoulder flexion with hands positioned at shoulder width) as follows: 1) Classic Quadruped (CQ): 180° shoulder flexion with external shoulder rotation and forearm in the neutral position. Contralateral hip extension up to 0° with maximum plantar flexion (Figure 1A); 2) Functional quadruped (FQ): Maximal extension of the upper limb and contralateral lower limb extension to 0° and maximum plantar flexion (Figure 1B); Homolateral quadruped (HQ): Same as the classic, but the homolateral lower limb and upper limb (Figure 1C). The subjects were familiarized with the exercises before data recording to ensure the perfect execution of each QE variation. Each QE was repeated three times for 10 s in each attempt, with 30 s of rest between attempts and an interval of 5 min before starting the following exercise to minimize the fatigue effects.

### Data recording

Oliveira et al. (1996) report that the stabilometric data follow a Gaussian distribution in the two directions investigated (x: latero-lateral; y: anteroposterior). Thus, the calculated elliptical CoP area (mm^2^) contained 95% of the samples (= 750) in both investigated directions, at 1.96 standard deviations in *x* and *y* from the dominant hand on the ground support.

According to the following equation, the temporal parameter extracted from the sEMG signal was the root mean square (RMS value).

$$\mathrm{RMS}=\sqrt{\frac{1}{N}\sum_{n=1}^{N}\mathrm{EMG}{\left[n\right]}^{2}}$$

where N represents the number of samples (= 10,000) in the analyzed intervals (T = 5 s).

The RMS values were normalized in relation to this parameter also obtained from sEMG signals recorded during isometric voluntary contractions (IVCs) derived from the two control tasks performed by the participants. It allowed comparing the muscle recruitment pattern of the four muscles studied in the three QE postures. One of the control tasks involved achieving a full lumbar spine extension for 10 s. The participant assumed the ventral decubitus posture, with the lower limbs attached to the stretcher, and kept his hands on the nape of the neck. To record the *transverse abdominis* muscle IVC, the participants performed a plank exercise on the elbows for 10 s, considering that this muscle has an activated function in the trunk stabilization in this position. In summary, the normalization of the myoelectric activity of each muscle (right and left sides) occurred from the ratio between the RMS value obtained from each QE posture and the two control tasks performed.

The sEMG signals extracted for analysis were those with a duration of 5 s but comprised between the initial and final 2.5 s from maintaining the final posture referring to each QE and the two control tasks. This procedure was adopted to guarantee the minimal stationarity of the sEMG signals. The CoP data for analysis was also obtained from the same time interval.

The QE were carried out under the supervision of one of the researchers. Upon reaching the final position of each posture, the participant should remain in it for 10 s, similar to the control tasks. The ordering in the execution of the three QE were done in a randomized way. At the end of each QE sequence, participants should rank the effort perceived by the modified Borg index (0–10; Borg, 1998) in each exercise. The data obtained from the second repetition of each QE were arbitrarily considered for analysis.

### Statistical analyses

Data were analyzed with custom-made scripts written in the R language (version 4.1.0, R Core Team, Vienna, Austria, 2021). Data normality was assessed by the Shapiro-Wilk test whenever necessary. A three-way analysis of variance (ANOVA) was adopted for sEMG data assessment (factors: *QE* × *Hemibody Side* × *Muscle*). In turn, the effect of the QE on the CoP was analyzed with a one-way ANOVA. Bonferroni *post-hoc* test was applied whenever necessary. The Friedman test was applied for the Borg scale data with Dunn as *post-hoc* test for multiple comparisons. The significance level adopted was set at 5% (*α* = 0.05).

## Results

Thirty healthy women (age: 22.1 ± 1.55 years old; height: 1.60 ± 0.06 m; body mass: 54.4 ± 9.02 kg; BMI <25 kg/m^2^; Oldfield’s score: +80.4 ± 33.8) participated in the study.

Regarding the Borg scale, when comparing the conditions tested, there was a statistically significant difference (*p* < 0.01, Kendall’s W = 0.03, 95%CI [−0.68 – 1.00]). Higher values were found for the BORG scale in HQ (4.35 ± 1.8) than in the other two QE (CQ: 2.1 ± 0.8; FQ: 2.9 ± 1.6; *p* < 0.05) (Figure 2).

**FIGURE 3:**
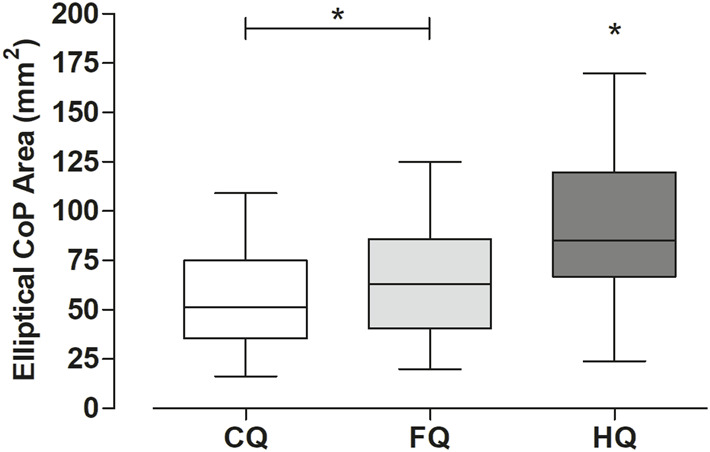
Results (medians and quartiles) of the elliptical CoP area (mm^2^) were obtained from the palmar support on the three conditions tested. The homolateral exercise (HQ) also resulted in significant (*p < 0.05) greater elliptical CoP areas in contrast to the other two (CQ and FQ).

In turn, for the elliptical CoP area, there was also a statistically significant difference between tested conditions (*p* < 0.01, η^2^ = 0.18, 95%CI [0.07–1.00]). HQ presented significantly greater areas than CQ (MD = 32.04) and FQ (MD = 25.15) (*p* < 0.05), as shown in Figure 3.

**FIGURE 4:**
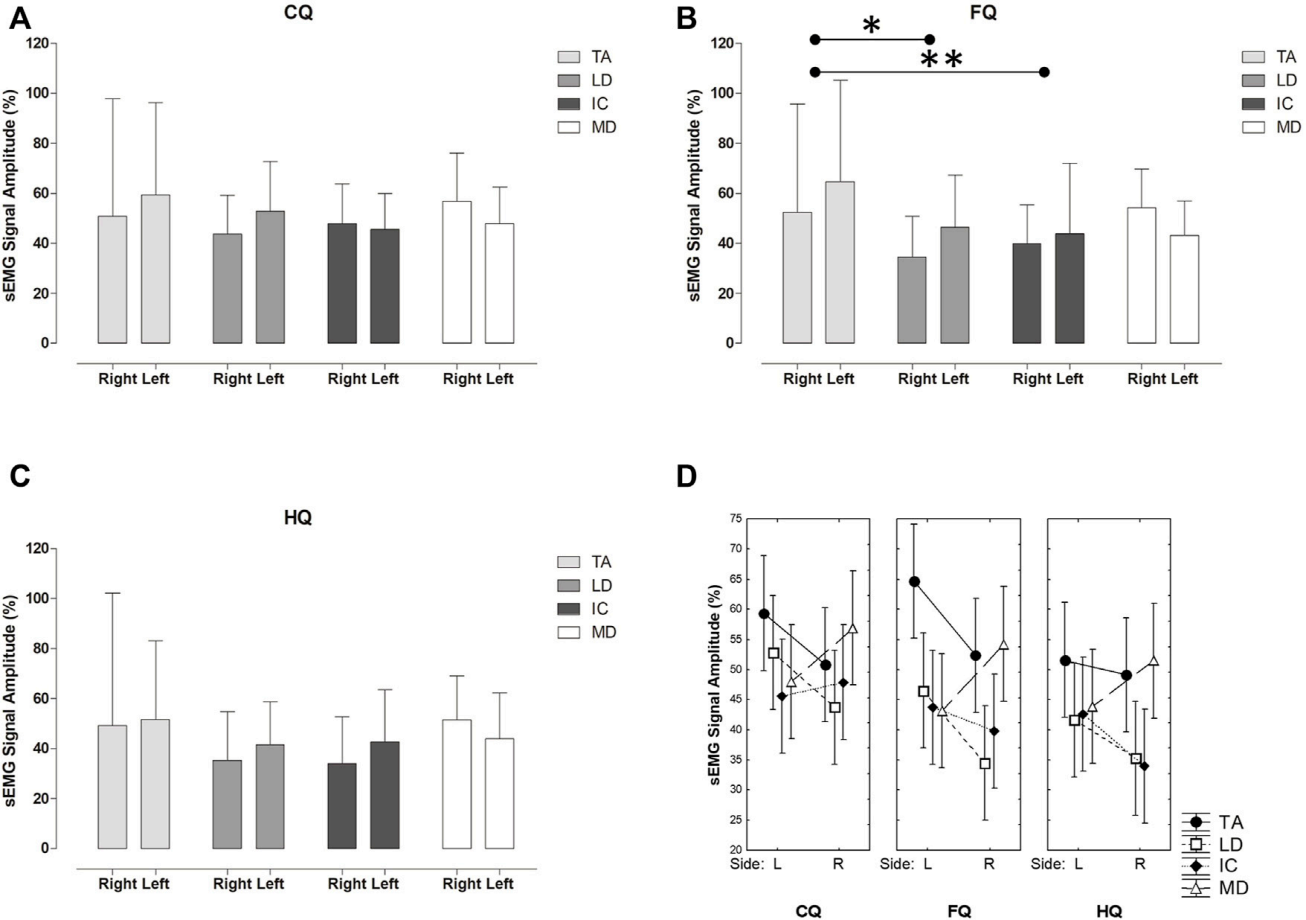
Mean (±SD) of relative sEMG signal amplitude (%) comparing the TA, LD, CI, MD muscles bilaterally for the **(A)** classic quadruped (CQ) **(B)** functional quadruped (FQ) **(C)** homolateral quadruped (HQ) and between the hemibody sides for the three postures **(D)**; *p = 0.01; **p = 0.03. Hemibody sides: L: Left; R: Right.

The relative sEMG signal amplitude presented a statistically significant difference for the main effects *QE* (*p* = 0.01, F_(2, 696)_ = 4.10, η^2^ = 0.01, 95% CI [0.00–1.00]) and *Muscle* (*p* < 0.01, F_(3, 696)_ = 9.26, η^2^ = 0.04, 95%CI [0.02–1.00]). There was no interaction between factors *QE* × *Muscle* (F_(6, 696)_ = 0.56; *p* = 0.76). Considering the *QE,* CQ was significantly greater in contrast to HQ (MD = 6.93, *t* = 2.86, *p* = 0.01) but not in relation to FQ (MD = 3.27, *t* = 1.35, *p* = 0.36) (Figures 4A–C). There was no statistically significant difference for the sEMG signal between sides (F _(1, 696)_ = 1.99; *p* = 0.15) (Figure 4D). Besides, there was no interaction among the three factors (*QE* × *Hemibody Side* × *Muscle*; F_(6, 696)_ = 0.41; *p* = 0.86).

## Discussion

The present study aimed to characterize three different QE postures commonly adopted in rehabilitation and functional training due to the lack of consensus regarding the subsidies supplied to trainers and physical therapists in prescribing these exercises. According to the results found, it is suggested that the three postures differ not only in the perceived exertion but also in the muscle recruitment pattern and postural control, which are discussed below.

### Borg scale

The results obtained from the modified Borg scale suggest that the HQ was more challenging for the participants than the other two postures. Like the classic quadruped *bird dog*, the starting position is under four supports—hands and knees. From this position, the subject performs shoulder flexion at 180^o^ while the homolateral lower limb extends in a 90^o^ motion to the neutral position. The support base, configured by the ground support points, is defined on the right side in this posture. Because the center of gravity is shifted to the left, there may be a left trunk rotation to project it into the support base. This hypothesis seems to be corroborated by the statokinesiogram, whose elliptical CoP area was significantly larger than CQ and FQ. Therefore, based on the modified Borg scale, it is suggested that the homolateral condition (HQ) seems more challenging than the other two postures.

Interestingly, it did not show a higher sEMG or asymmetrical signal amplitudes compared to the CQ and FQ postures. Hence, although the homolateral exercise was advocated by Rudolph Klapp and described as an effective exercise for scoliosis treatment (Lunes et al., 2010), we may conjecture that HQ may not be as suitable as hypothesized in such a population based only on the sEMG signal amplitude. It means that if someone aims to increase the muscle recruitment of one side of the trunk concerning the other to correct any postural deviations, HQ may not be the best exercise. Thus, trainers and physical therapists must carefully interpret the sEMG signal amplitude as a parameter when prescribing QE.

### Elliptical CoP area

According to our literature review, no other studies had investigated the effects of QE postures on CoP excursion areas. Accordingly, it was possible to enlighten some of the strategies adopted by the participants in the balance control in each of the QE postures tested.

As previously highlighted, the homolateral condition (HQ) seemed to lead to a more challenging task and, therefore, unstable than the classic control condition (CQ), corroborated by the modified Borg scale. The elliptical CoP area of HQ toured the anteroposterior and mid-lateral axes more significantly than CQ, which may be related to the homolateral disposition of the upper and lower limbs in this posture. We must mention that we hypothesized that a reduced CoP area would be accompanied by increased sEMG signal amplitude. It would mean that the smaller the displacement of the CoP, the greater the muscle recruitment to make the body more rigid in facing the challenges in postural control of each QE. However, we did not observe such an agreement, which suggests that there seems to be no association between the parameters investigated from the statokinesiogram and the sEMG signal.

Moreover, we highlight the impossibility of recording the statokinesiograms of the entire support base in all three postures. This measurement would make it possible to understand better how the body’s centre of gravity behaves as a result of the tasks performed.

### The myoelectric activity

Graham (2009) described CQ as an exercise to recruit the core, triggering the trunk, abdomen, hip, and shoulder girdle muscles. Consequently, the CQ can be considered the most helpful exercise to stimulate spinal stabilization within clinical practice in offices and gyms. Balanced muscle recruitment was found on both the rising hand and the palmar support sides. The sEMG signal found in the data suggests that this is an exercise for strength development, i.e., above ∼41% of the IVCs in all evaluated muscles (Ekstrom et al., 2007). Besides the muscle recruitment pattern found in the present study, this QE posture suggests a more remarkable and balanced muscle activation between the sides than FQ and HQ. It is appealing since the posture adopted during the exercise is asymmetrical and provides lower modified Borg scores. In this sense, our results did not corroborate García-Vaqueiro et al. (2012), who observed greater activation in the left TA (referring to the rising hand). Some hypotheses were raised regarding the divergences between the results observed in the present study and those reported by García-Vaqueiro et al. (2012). Primarily, the participants were instructed to perform the two control tasks that contributed to the normalization process of the sEMG signal without other resistive forces besides those related to the body parts’ weight. Additionally, the RMS value, in contrast to the rectified mean value, seems to offer advantages in interpreting muscle recruitment mechanisms since the former is related to the power of the sEMG signal (De Luca, 1997). Besides, although the authors report minimal differences between men and women regarding the electromyographic pattern and have made their analyses based on both groups, our study sample presented more homogeneous characteristics. Accordingly, some methodological issues may have contributed to these different findings, which deserve further clarification in future studies.

Comparing FQ with the CQ, where the change was only based on shoulder movement, being extension rather than flexion, the activation of the TA and MD muscles was similar. In addition, we observed that the IC and LD activations showed a minor statistically significant difference in the functional quadruped compared to the QC.

As previously described as an exercise for scoliosis treatment (Lunes et al., 2010; Dantas et al., 2017), HQ provided higher instability and Borg scores. Interestingly, they did not result in greater levels of muscle recruitment. Despite a greater but not significant activation in the TA concerning the other muscles, the larger supporting side with more evident recruitment of MD, LD, and IC muscles presented a lower activation than the previous exercises. The greater CoP excursion area in the supporting hand seems to have led to a less active muscle strategy to make the balance condition more flexible. Future studies relating the postural instability and the degree of muscle activation of a given exercise may benefit the assertive exercise prescription.

Regarding palmar stability and perceived exertion, HQ was the most challenging exercise. However, as previously mentioned, HQ did not show greater muscle activation than the others. These results reinforce that interpreting the contribution of intervening variables of QE exercises solely by the magnitude of muscle contraction may be misconceived. Based on the sEMG signal amplitude, CQ seemed more symmetrical in muscle recruitment among the studied muscles bilaterally, most likely because the posture adopted leads to a projection of the centre of gravity to the centre of the support base. However, this is only a conjecture since we could only monitor the CoP excursion area from only one of the points of the base of support. Even so, our findings suggest that CQ can be an excellent option for the bilateral recruitment of the muscles responsible for spine stabilization. Therefore, from the present results, we believe in having found some hints that should be considered by health professionals in the process of gradation of QE exercises.

Finally, we state that: 1) The muscular recruitment of the investigated muscles does not seem to follow the level of perceived effort in the three different studied postures of quadrupedal exercises; 2) The classic posture of the quadrupedal exercises seems to offer muscle recruitment that, in addition to being greater than the other two postures studied, also proved to be more symmetrical between the sides; and 3) Given the characteristics of perceived effort, the challenge in postural control, and muscle recruitment, it is suggested that each of the postures assumed may be more clearly adopted by different subjects with different demands/capacities. We may conclude that it is possible to prescribe these conditions with greater assertiveness and safety from these data.

## Data Availability

The raw data supporting the conclusion of this article will be made available by the authors, without undue reservation.
